# 3D Plasmon Coupling Assisted Sers on Nanoparticle-Nanocup Array Hybrids

**DOI:** 10.1038/s41598-018-19256-7

**Published:** 2018-02-14

**Authors:** Sujin Seo, Te-Wei Chang, Gang Logan Liu

**Affiliations:** 1 Department of Materials Science and Engineering, University of Illinois at Urbana-Champaign, Urbana, IL 61801 USA; 2 Department of Electrical and Computer Engineering, University of Illinois at Urbana-Champaign, Urbana, IL 61801 USA; 3 Micro and Nano Technology Laboratory, University of Illinois at Urbana-Champaign, Urbana, IL 61801 USA

## Abstract

Unique colorimetric optical properties of nanomaterials can effectively influence the light absorption or emission of molecules. Here, we design plasmonic substrate for surface-enhanced Raman scattering (SERS) by inducing three-dimensional (3D) hot spots on the sensing surface. The 3D hot spots are formed by the self-assembly of plasmonic nanoparticles (NPs) on a 3D plasmonic nanocup array structure. This 3D hot spot formation on the periodic nanocup arrays achieves much higher SERS enhancement factor than the 2D NP arrays, which have been conventionally sought SERS substrates. We also utilize the colorimetric properties of the nanocup arrays for an additional degree of SERS enhancement. Colorimetry, achieved by tunable plasmon resonance wavelength by controlling dielectric property on the nanocup array surface, eases the modulation of the plasmonic resonance condition without modifying the nanostructure design. By continuously monitoring the shifts of the plasmon resonance condition and its effect on the light absorption and emission of the nearby molecules, we verify that larger SERS enhancement is achieved when the plasmon resonance wavelength is matched with the Raman excitation wavelength. The ease of plasmon resonance tuning of this nanocup array-nanoparticle hybrid structure allows versatile SERS enhancement for a variety of different Raman measurement conditions.

## Introduction

The signal amplification without losing signal-to-noise ratio is critical in sensing and detection. The discovery of the strong electric field confinement and scattering phenomena, or so-called plasmonic effects, near the surface of metallic objects in nanoscale has birthed the field of surface enhanced Raman scattering (SERS)^1^. As a result, the intrinsically weak Raman signal, which has been underutilized in the sensing field, is now actively used to obtain unique molecular information with its amplified signal near plasmonic surface. The advantage of using SERS for sensing target analytes is the direct quantification without optical labels (e.g., fluorophores and chromophores), which requires additional steps with specific labeling or enzymatic chemistries^2^. Its high specificity allows for detections even in complex media (e.g., cell culture media, blood serum, or buffer solutions)^3–5^. Furthermore, there is no photobleaching problem unlike fluorescence^6^. With these advantages, the advances of SERS technologies by tuning the plasmonic properties of the sensing surface have accomplished detections of cancer biomarkers^4,7^, glucose^8^, infection related disease biomarkers^9,10^, drugs or even pesticide residues on fruits^11^.

Many plasmon-based SERS substrates were fabricated using Ag rather than Au due to larger SERS enhancement factor. As it has more damping of plasmon oscillation by having interband transition in the visible light range, Au typically shows smaller SERS enhancement factor than Ag^12,13^. However, Ag is prone to oxidation in high electrolyte concentrations^14,15^. The use of Ag SERS substrates therefore requires special caution when detecting target in high salt solution. Due to its instability, many Ag-based SERS sensors are not re-usable or recyclable. In addition, strong SERS resulted from the Ag plasmonic surface is sometimes under suspicion of having oxidation of Ag during sensing, resulting in reduced Ag size or shape change and exhibiting varying SERS results over time^16–18^.

In order to utilize the SERS signal for sensing the target in complex media or in high electrolyte conditions, it is ideal to have plasmonic SERS substrates consisted of relatively more stable materials than Ag. When using Au for SERS-active plasmonic substrate fabrication, optical engineering through surface treatment is needed for compensating the low SERS enhancement factor compared to Ag. Here we performed the three-dimensional (3D) hot spot engineering to improve SERS effects, while keeping the stability and reliability of the Raman measurement. We introduced the plasmonic Au spherical nanoparticles (NPs) on the 3D nanocup array structure with plasmonic effects for tuning the number and the density of the hot spots. While many researchers have been mainly focused on the two dimensional (2D) NP arrays for an optimum SERS substrate design, we utilized a periodically debossed film with nanocups as a template for a uniform 3D NP assembly and thus achieved 3D plasmon coupling on this substrate. Stronger SERS enhancement factor was observed from the large number of 3D plasmon coupling among plasmons from NPs and the nanocup arrays than from the 2D NP arrays, since denser hot spots were resulted with strong electric field confinement^19–21^.

The minimum requirement for achieving SERS is the presence of plasmons near the sensing surface. The degree of SERS enhancement factor, on the other hand, is closely related to how close the plasmon resonance condition is to the SERS detection condition. Raman signal can be amplified by strong excitation energy. Since strong electric field confinement and scattering is observed near the plasmon resonance condition^22^, one can achieve additional SERS enhancement by matching the plasmon resonance with the excitation source wavelength. Similar study was conducted using a paired chromophore near the plasmonic object to achieve higher SERS performance^18^. When the plasmon resonance wavelength is matched with the excitation laser wavelength, more photons are involved in the excitation of the molecular vibration and stronger Raman scattering is resulted. Most SERS substrates have been specifically designed to achieve their plasmon resonance conditions matched with the Raman excitation wavelengths (e.g., 785 nm). The main drawback of such nanostructure engineering is the limitation of the measurement condition, as detections of some target molecules require different laser excitation energy or wavelengths.

In order to sense targets with various Raman detection conditions (e.g., various excitation laser wavelengths), a SERS substrate with easily tunable plasmon resonance condition is in need. In this article, a plasmonic substrate that is composed of nanocup arrays is implemented as a versatile, effective, and stable SERS sensor. One of the characteristic plasmonic effects induced by the nanocup arrays is the colorimetric property: a transmission or a reflection peak, located in the visible light range, shifts with the plasmon resonance condition change and therefore the corresponding color of the device surface changes in transmission or in reflection mode^22^. The plasmon resonance condition sensitively changes when the surrounding refractive index (RI) on the sensing surface varies^22,23^. This consequently provides an extra degree of freedom for tuning the SERS performance by using this colorimetric property of the nanocup arrays. In other words, SERS enhancement factor (EF) can be further improved by matching the plasmon resonance wavelength to the Raman excitation source wavelength through the RI variation on the sensing surface. Using this colorimetric nanocup array substrate, a so-called nano-Lycurgus cup array (nanoLCA) substrate^23,24^, we demonstrate a tunable SERS EF by manipulating the sensing media on this sensing substrate.

**Figure 1:**
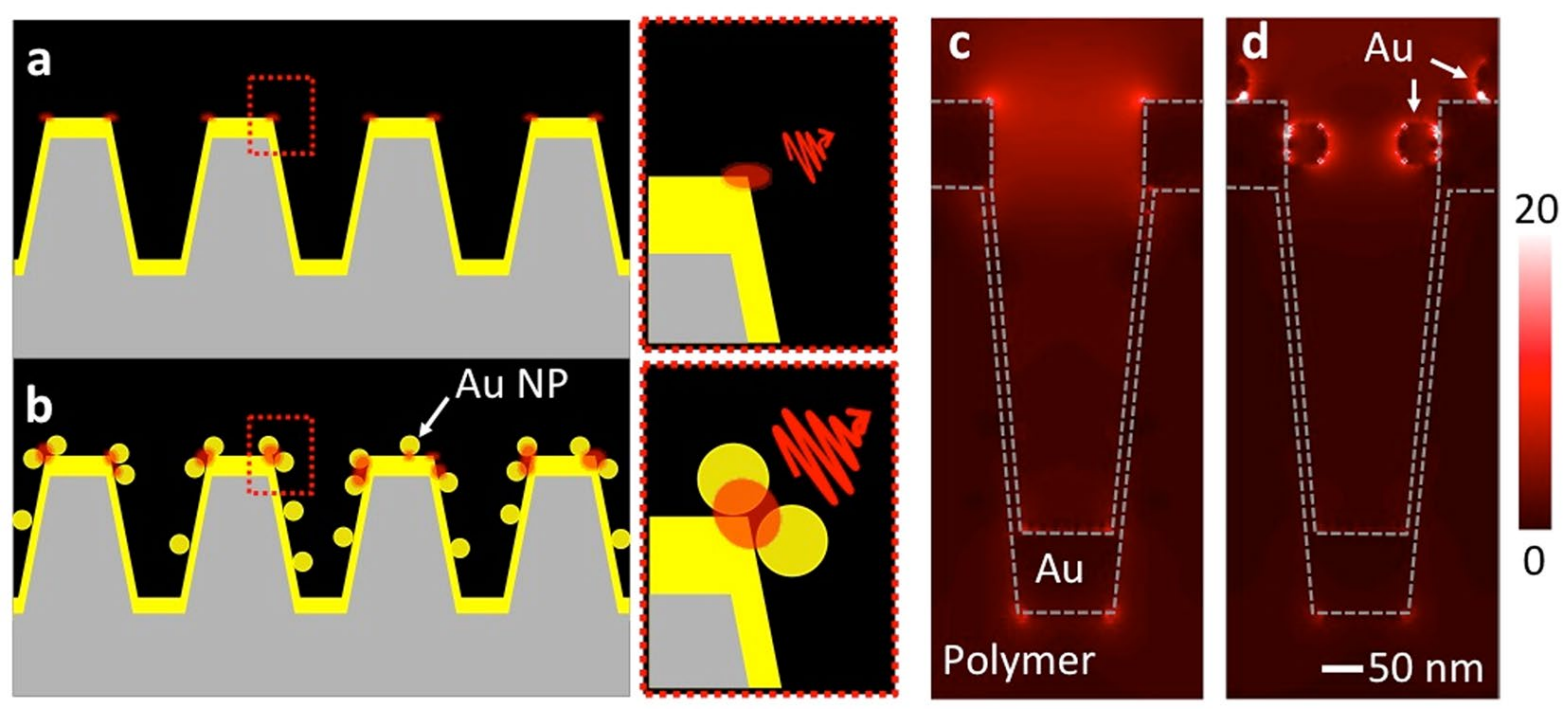
(**a**,**b**) Schematic representation of the SERS enhancement mechanism on the nanoLCA and the NP-nanoLCA. (**b**) Denser hot spot formation by self-assembling NPs on the colorimetric plasmonic substrate drives larger SERS EF. 3D-FDTD simulated electric field distributions (|E|) on (**c**) the nanoLCA and (**d**) the NP-nanoLCA show that the NPs attached in the nanocup effectively confine the electric field near the surface of the nanocup.

Although the nanoLCA itself shows good SERS performance, larger improvement was achieved by adding Au NPs on the Au nanocup arrays. It was accomplished by forming denser hot-spot generation when the plasmonic NPs were assembled on the plasmonic sensing surface. The idea of the additional SERS improvement by the NP assembly on the nanoLCA is shown in the schematic illustration (Fig. 1a,b). A quick verification of this idea was demonstrated using the finite-difference time-domain (FDTD) method (Fig. 1c,d): stronger electric field was confined near the surface of sensing surface, when the NPs are assembled on the nanoLCA.

## Results

### Fabrication and optical properties measurement of hybrid NP-nanocup array structure

**Figure 2:**
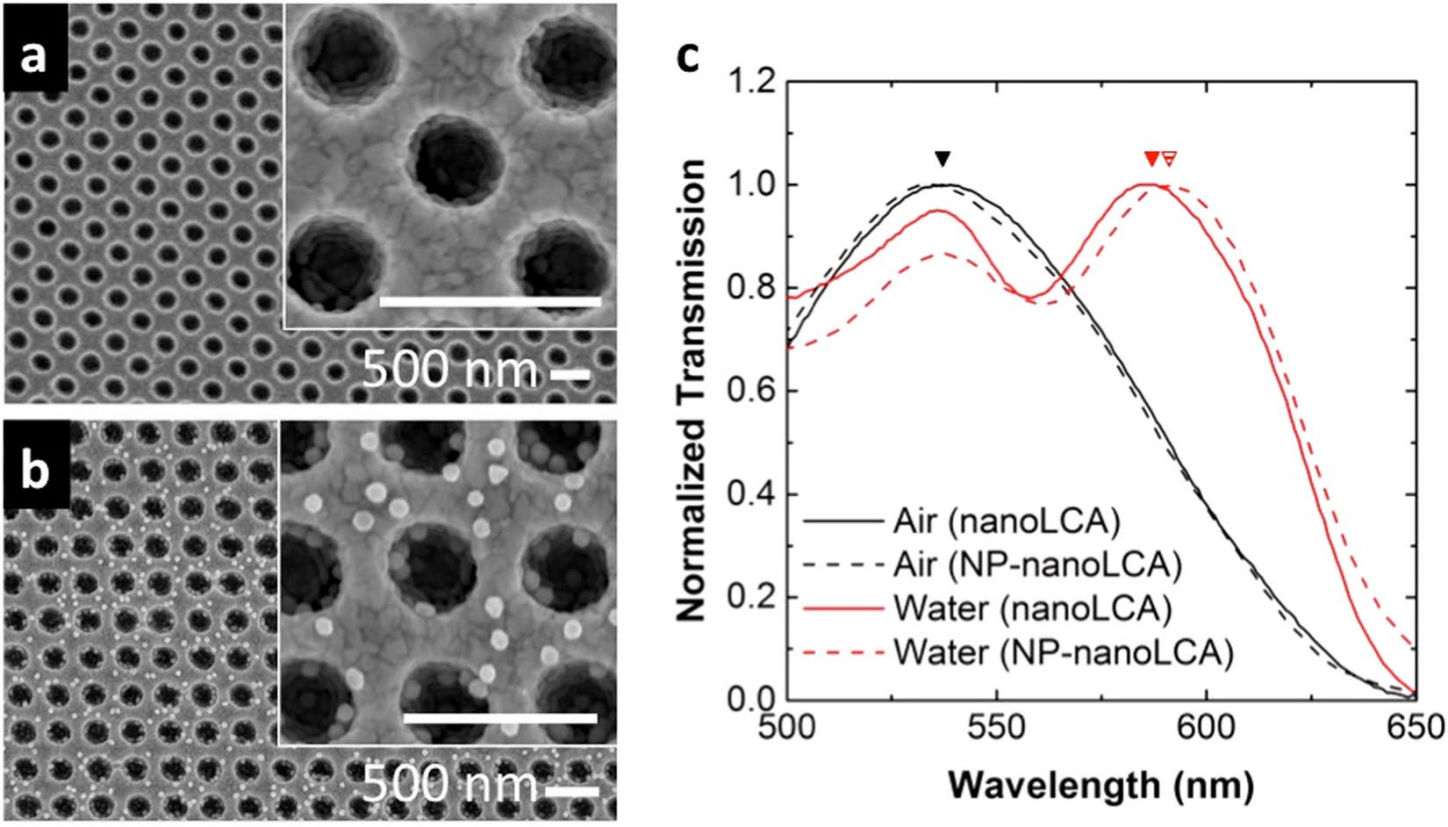
SEM images of (**a**) the regular nanoLCA and (**b**) the NP-nanoLCA. (**c**) Transmission spectra of nanoLCA and NP-nanoLCA with the surrounding media of air and water.

The nanoLCA and the NP-nanoLCA were prepared to evaluate the SERS performance. The nanoLCA substrate was fabricated by the replica molding technique with a mold, which is composed of nanopillar arrays. The replica molding process using UV-curable polymer produced the polymeric nanocup array structure. The subsequent deposition of thin Ti (9 nm) and Au (90 nm) layers on the polymeric nanocup array structure achieved the plasmonic properties of the nanoLCA substrate^23^. The surface topology was characterized by the scanning electron microscopy (SEM) (Fig. 2a). The NP-nanoLCA substrate was prepared after fabricating the nanoLCA. Cysteamine, which has both amine and thiol functional groups at each end, was chosen as a linker molecule in order to electrostatically attract negatively charged NPs to the nanoLCA surface^20,25^. After treating the nanoLCA surface with cysteamine, 50 nm Au NPs were assembled by incubating for 24 hours. This resulted in self-assembled monolayer (SAM) of NPs on the nanoLCA substrate, forming the NP-nanoLCA^20^. The SEM image of the NP-nanoLCA in Fig. 2b shows the SAM of NPs on the nanoLCA substrate. The average number of NPs was 4.26 NPs per nanocup (see Supplementary Fig. S1).

**Figure 3:**
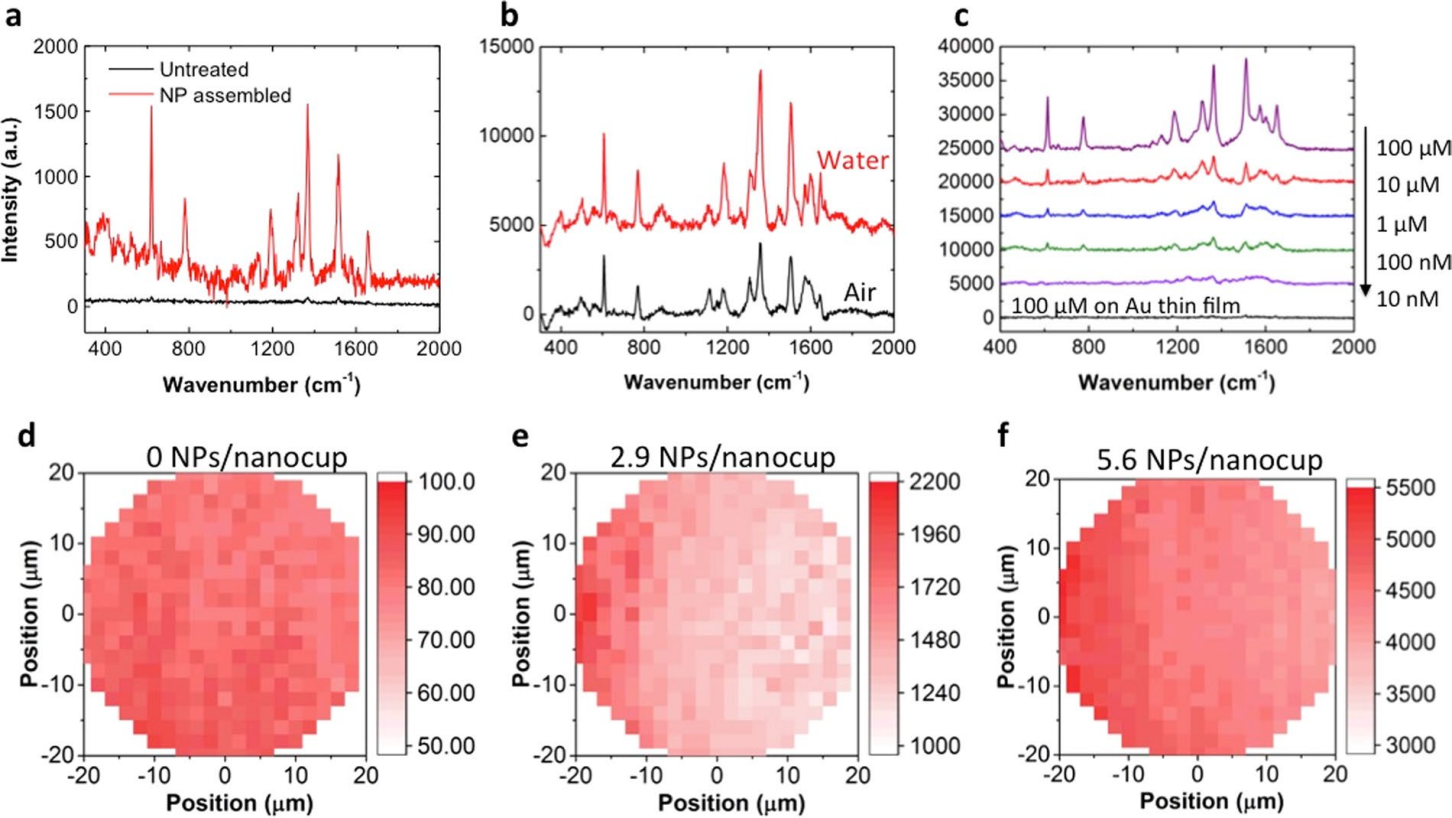
(**a**) Raman spectra of 100 μM R6G on the nanoLCA (black line) and the NP-nanoLCA (red line) measured with an exposure time of 1 sec. (**b**) Raman spectra of 100 μM R6G on the NP-nanoLCA measured in air (dry state, black line) and in water (wet state, red line) with an exposure time of 10 sec. (**c**) Raman spectra of R6G in different concentrations on the NP-nanoLCA, measured in the wet state. (**d**–**f**) 2D Raman intensity map of 100 μM R6G on the NP-nanoLCA with varying NP numbers per nanocup. The intensity at 1357 cm^−1^ Raman peak was plotted and the spectra were measured in dry state.

The surface plasmon resonance wavelength of the nanoLCA is equivalent to the transmission peak, since the nanoLCA has strong extraordinary transmission phenomena at its plasmon resonance^24,26,27^. As Fig. 2c shows, the plasmon resonance wavelengths of the nanoLCA in air and in water were 536 nm and 586 nm, whereas those of the NP-nanoLCA were 533 nm and 592 nm. The transmission peaks marked with the inverted triangles represent the localized surface plasmon resonance modes that are critical in SERS and electromagnetic field scattering. The study on the plasmon resonance mode of nanoLCA and NP-nanoLCA is described in Supplementary Fig. S2. Although there were only 3 nm and 6 nm differences in the resonance wavelengths in air and in water between the nanoLCA and the NP-nanoLCA, the Raman scattering intensity was much larger on the NP-nanoLCA than on the nanoLCA (Fig. 3a). The Raman scattering from 10 μM rhodamine 6 G (R6G) was measured on the nanoLCA and the NP-nanoLCA with the integration time of 1 sec and the laser power of 120 μW. When NPs were assembled on the nanoLCA structure, the intensity of all characteristic R6G Raman peaks (i.e., 607.2, 770, 1177, 1308, 1357, 1504, 1572, 1644 cm^−1^) was increased. The intensity of the Raman peak at 1357 cm^−1^, which represents the vibrational mode of aromatic C-C stretching^28^, was used to calculate the EF. The increment in the peak intensity by 54.74-fold was observed after assembling NPs on the nanoLCA in Fig. 3a.

The addition of the plasmonic Au NPs on this SERS substrate (i.e., nanoLCA) resulted in the formation of denser hot spots on the sensing surface and hence greater SERS performance was achieved. Although the plasmon resonance wavelength of each nanoLCA and NP-nanoLCA was close to each other, the NP-nanoLCA had more than 50-fold of SERS enhancement. This implies that the density and the number of hot spots on the same sensing area are critical to the SERS enhancement.

We note that both the integration time of 1 sec and the laser power of 120 μW used in this experiment are relatively small, compared to other SERS reports; the integration time between 10 sec and 30 sec has been commonly used for SERS measurements with a mW range laser power^29–31^. This indicates that the nanoLCA and the NP-nanoLCA allow cost-effective and energy-efficient Raman measurements due to their strong light scattering properties. In addition, the short integration time during the Raman measurement can avoid melting or deformation of the underlying sensing surface, especially when the substrate is made up of polymer below the top metallic layer.

### Tuning plasmon resonance through colorimetry for an optimum SERS EF

Strong scattering electric field from the metallic surface of a nanomaterial is observed at the plasmon resonance wavelength. The SERS is thus enhanced when the resonance wavelength is closer to the laser excitation wavelength, since more photons can be involved in the molecular excitation process. The SERS enhancement by matching plasmon resonance with the excitation laser wavelength has been reported^32,33^. Most reports demonstrated fabricating new substrate structures in order to design the plasmon resonance placed at a specific wavelength (i.e., Raman excitation wavelength). In contrast to these reports, the use of the colorimetric plasmonic surface as a SERS substrate can avoid extra substrate designs and fabrications by allowing easy control of the plasmonic resonance conditions, which effectively saves extra time and cost associated with substrate re-design. The plasmon resonance of a colorimetric sensor is easily tuned by controlling the surrounding RI^34^; thus, a single substrate with tunable resonance wavelength can be used in a variety of different Raman conditions with high SERS EFs.

Considering the nanoLCA’s colorimetric properties, or the gradual transmission color change due to the red-shift of its plasmon resonance wavelength with increasing surrounding RI, Raman measurements on nanoLCA in air (1 RIU, or in dry state) and in water (1.33 RIU, or in wet state) are expected to result in different SERS EFs. In order to find an optimum SERS condition on the colorimetric plasmonic substrate, we examined the relationship between the plasmon resonance wavelength and the Raman measurement condition, such as laser excitation wavelength (e.g., 532 nm or 632 nm). The surface plasmon resonance wavelength of nanoLCA in air was closer to the laser wavelength of 532 nm, whereas the plasmon resonance in water was closer to the laser wavelength of 632 nm. We verified that the R6G Raman scattering in air was larger with 532 nm laser source than the measurement with 632 nm laser (see Supplementary Fig. S3). Larger SERS enhancement with 532 nm light source is resulted by larger amount of scattered light from the device surface near the surface plasmon resonance wavelength.

The R6G Raman measurement is mainly performed at 632 nm. This is because the R6G molecules emit fluorescence when excited at 532 nm. Therefore, the ideal SERS substrate for measuring the R6G Raman should have the surface plasmon resonance wavelength close to 632 nm. We took the advantage of colorimetric properties of the nanoLCA substrate by tuning the surface plasmon resonance to locate near 632 nm. This was accomplished by the control of the interfacial dielectric properties on the surface. As verified in the previous experiment, the nanoLCA has the surface resonance wavelength of 586 nm with water interface, which becomes closer to the 632 nm laser excitation wavelength. We characterized the SERS performance of nanoLCA and NP-nanoLCA substrates with this shifted surface plasmon resonance condition and the 632 nm excitation laser source.

As Fig. 3b shows, we confirmed that both nanoLCA and NP-nanoLCA had larger SERS enhancement in wet state when compared to the dry state measurement. The nanoLCA had an average of a 2.16-fold and the NP-nanoLCA had a 2.17-fold of enhancement (see Supplementary Fig. S4). Similar SERS EFs on nanoLCA and NP-nanoLCA were achieved, because the amount of surface plasmon resonance wavelengths shifts of both substrates was comparable when the surrounding media changed from air to water (i.e., 586 nm and 592 nm for nanoLCA and NP-nanoLCA). Large SERS EF in wet state implies that the nanoLCA or the NP-nanoLCA can be very useful for biosensing, which typically involves buffer solutions, electrolytes, or cell culture media. As the direct target analysis without drying the solvent on the sensing surface is more favorable for those biomedical applications, stable Au-based nanoLCA or NP-nanoLCA with colorimetric properties can be a great SERS substrate choice.

The light absorption properties of a molecule are sometimes susceptible to the variations in dielectric properties of surrounding media. The light absorption of R6G was reported to decrease with increasing amount of water present in air^35^. If less light is absorbed by the molecules, the Raman scattering is also likely to decrease; however, more than twice the Raman scattering intensity was observed on the plasmonic nanoLCA substrate when measured in the wet state. This indicates that the SERS effect by the plasmons dominates over the intrinsic molecular optical property change by providing larger molecular excitation.

Raman spectra of increasing R6G concentrations were measured on the NP-nanoLCA with the water surrounding (Fig. 3c). The lowest detectable concentration was 100 nM. As we put 2 μL R6G solution (100 nM) over the area with 1 mm radius, the density of 63.66 fmol/mm^2^ is obtained. When we consider the actual number of R6G molecules under the laser spot size of 6.06 μm with 20 × objective lens, the Raman signal was collected from 1.836 attomol R6G. Therefore, the NP-nanoLCA serves as a good SERS platform that can detect down to attomol range. When considering the number of R6G molecules per nanocup (the periodicity is 350 nm), approximately 4,696 R6G molecules per nanocup were detected on the NP-nanoLCA.

Although the 100 nM R6G may seems high in concentration for a SERS measurement, the detection was done by dropping only 2 μL of 100 nM R6G and measured with a few hundred-microwatt range of laser power. The volume required for this sensing was extremely small compared to other SERS studies. For example, S. Si *et al*.^36^ detected 0.1 nM R6G by using Au monolayer film, but the volume they used was 100 μL. Another study by Y. Liu *et al*.^37^ reported the SERS measurement of ~2 nM R6G by dropping 10 μL and measured with 90 mW laser power, which is 750 times higher than the power we used to excite the Raman (i.e., 120 μW). Thus, the NP-nanoLCA is well suited to the applications for portable SERS-based chemical detections, which can effectively work with limited amount of samples and low power.

### Effect of the hot spot density

R6G Raman intensity map was plotted by measuring Raman peak intensity at 1357 cm^−1^ on the NP-nanoLCA substrates. Three substrates with different average number of NPs per nanocup were prepared and characterized in this experiment (Fig. 3d–f). The average number of 2.9 NPs/nanocup on the nanoLCA was obtained by incubating the NP solution on the cysteamine-nanoLCA substrate for 16 hours and 5.6 NPs/nanocup was achieved by incubating the NP solution for 48 hours. Despite the semi-random assembly of NPs on the nanoLCA structure, uniform SERS over a large area was obtained, especially when the NP-nanoLCA had 5.6 NPs/nanocup. Since approximately 230 nanocups were exposed under a single radiation of the laser light, the resulting Raman signal was the averaged SERS performance of the 230 nanocups and this explains the uniformity of the SERS performance on the NP-nanoLCA.

The full Raman spectra from 20 different spots in 5 mm by 5 mm region also verified the signal uniformity (see Supplementary Fig. S5). This satisfies a requirement of a good and reliable SERS substrate candidate, which is the uniform SERS performance over a large area. This uniform performance is achieved by the underlying top-down fabricated periodic 3D structure (i.e., the nanocup arrays). Unlike the conventional colloid-based 2D SERS substrates, this top-down fabricated plasmonic substrate with periodic nanostructure exhibits much more uniform optical properties and serves as a platform for the uniform plasmon coupling. Inducing macroscopically uniform plasmon coupling with strongly confined electric field among the self-assembled plasmonic NPs and the underlying plasmonic nanostructure is, therefore, an effective way to enhance SERS on this nanoLCA substrate and is applicable to many other existing SERS substrates.

**Figure 4:**
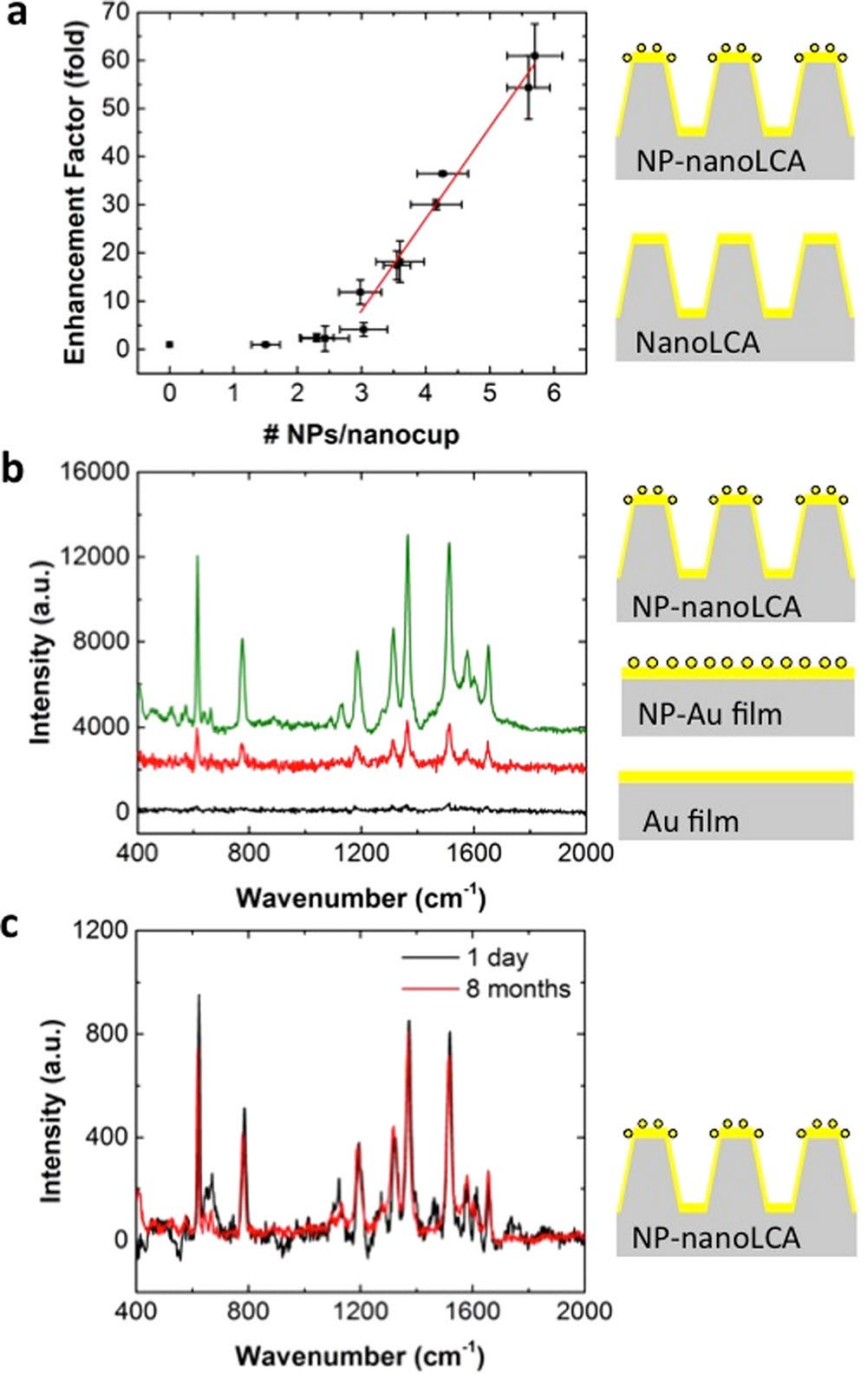
(**a**) SERS Enhancement factor from the NP-nanoLCA with 0~5.9 NPs/nanocup, calculated by comparing the Raman intensity on the nanoLCA. The error bars represent standard deviations. (**b**) 100 μM R6G Raman spectra measured on a Au film, NP-Au film, and NP-Au nanoLCA. The integration time was 10 sec. (**c**) 100 μM R6G Raman scattering measured after 1 day and 8 months from the NP-nanoLCA fabrication. The integration time was 1 sec.

As briefly mentioned in the previous paragraphs, the number of NPs assembled in each nanocup can be tuned by varying the incubation time of the NP solution during the NP assembly procedure. The enhancement factor was calculated by comparing the Raman intensity peak intensity at 1357 cm^−1^ between the nanoLCA, which has 0 NP/nanocup and the NP-nanoLCA. As Fig. 4a shows, a linear response of the Raman intensity was observed as more NPs were assembled on the nanoLCA substrate. The correlation was strongly found when more than 2.9 NPs were assembled per each nanocup. The average maximum SERS enhancement was 60.94-fold when the average of 5.7 NPs/nanocup were assembled on the nanoLCA substrate. The enhancement factor on this NP-nanoLCA was larger than that from the R6G detection using the commercial Klarite SERS substrate (see Supplementary Fig. S8).

If more NPs are assembled on the same nanoLCA substrate, the SERS EF is expected to increase more based on the extrapolated prediction of the fitted line in Fig. 4a. Two main approaches can be considered to improve the NP density on the nanoLCA: (1) increase the linker molecule’s density on the nanoLCA and (2) increase the concentration of the NP solution for the assembly. The concentration of the cysteamine molecular solution we used was 10 mM, which is equivalent to 6.023 × 10^6^ molecules in 1 μm^3^ volume. In contrast, the concentration of the NP solution was 2.0 × 10^11^ particles/mL, which is equivalent to only 0.2 particles in 1 μm^3^ volume. Therefore, if one uses more concentrated NP solutions for the NP self-assembly, stronger SERS enhancement will be feasible by obtaining denser hot spots on the sensing surface.

### 3D plasmon coupling effect

Two-dimensional (2D) arrays of plasmonic Au or Ag NPs, assembled on a flat surface, have also shown good SERS performance^36,38^. However, the SERS performance from the 2D NP arrays is solely dependent on how closely the NPs are arranged among them, or the quality of the NP assembly^19,39^. Unlike these 2D NP array SERS substrate, the use of underlying plasmonic nanostructured substrate produces 3D NP arrays and it supports much stronger localized surface plasmon effects due to additive optical coupling at the extremely small separation between two plasmons, one from the assembled NP and the other from the nanostructured substrate. The gap between the NP and the substrate is mostly governed by the length of the linker molecules that enable the NP assembly. The separation between the assembled NPs and the underlying plasmonic substrate is in sub-1 nm range. Thus, even though the NPs are not assembled close to each other in 2D, the strong electromagnetic field confined at a small gap between the NP and the plasmonic nanostructure enables SERS amplification.

Since the self-assembly of bottom-up synthesized spherical nanoparticles through the surface chemistry can form the uniform sub-1 nm gap between two plasmonic surfaces, this fabrication method is suitable for the SERS application. Some research papers demonstrated fabrication of the nanoparticle-like structures through the physical vapor deposition or the electrochemical deposition^40,41^. These methods enable fabricating such structures in a single step; however, the resulting nanoparticle structures lack the uniform separation between the underlying substrate and the additionally deposited nanoparticle structures. Thus, uniform and well-controlled plasmon coupling and hot spots are hardly achieved by these methods. In addition to the uniform plasmon coupling effects, the NP self-assembly also achieves 3D coupling effect along the 3D surface of the underlying structure.

In order to assess the influence of the underlying plasmonic substrate combined with the assembled NPs, the Raman scattering were measured and compared from three different substrates: Au film, Au film with NP SAM, and Au nanoLCA with NP SAM. The film substrates were prepared by depositing the same thickness (i.e., 90 nm) of Au on a glass slide. Figure 4b shows the Raman scattering of 100 μM R6G from each substrate. The NP assembled Au film showed 6.28-fold larger intensity at the Raman peak of 1357 cm^−1^ than the Au film did. In comparison, there was 24.58-fold enhancement when the NPs were assembled on the nanoLCA instead of the film. This implies that the extra plasmon coupling through the NP assembly on the plasmonic nanostructure adds stronger and denser hot spots, which eventually amplify the Raman scattering. In addition, the assembly of NPs on a 3D structure induces more hot spots exposed to the Raman probe molecules; thus, better SERS performance was achieved.

The stability of the SERS activity is one of the critical properties for a SERS sensor. We verified that the SERS effect of the NP-nanoLCA did not degrade much over eight months (Fig. 4c). The R6G Raman scattering was measured on the same NP-nanoLCA substrate at different times. One set of measurements was done after one day from the NP-nanoLCA’s fabrication and the other set of measurements was done after eight months from the device fabrication. 100 μM R6G solution was dropped on clean surface and dried right before each measurement. The measurements were performed with the same laser power (120 μW) and the integration time of 1 sec. Each spectrum represents the averaged values measured from five different spots. After eight months, 5.25% decrease in the characteristic peak intensity was observed. This degree of intensity variation, however, is in the range of the standard deviation of each set of spectral measurements; thus, the NP-nanoLCA can be considered as a stable SERS substrate.

**Figure 5:**
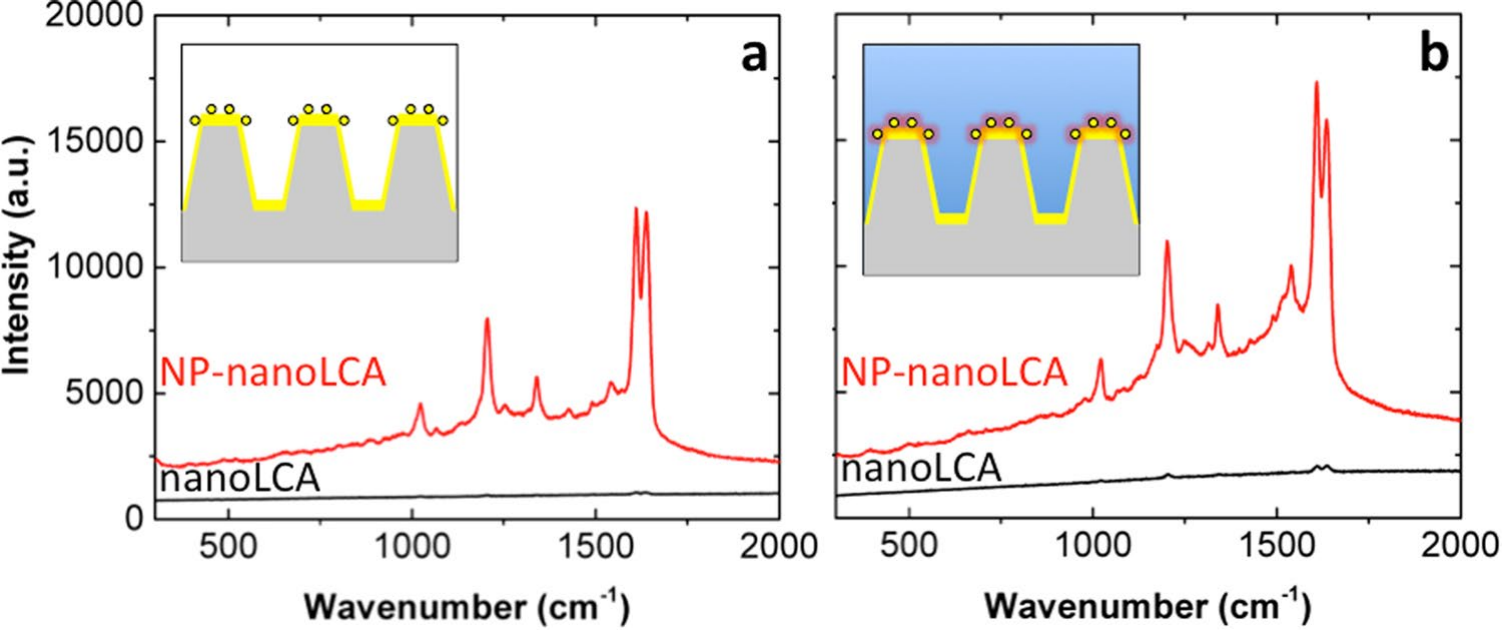
Raman spectra of BPE measured on the nanoLCA and the NP-nanoLCA. The spectra were measured (**a**) in air and (**b**) in water.

The SERS performance of nanoLCA and NP-nanoLCA substrates was characterized with trans-1,2-bis(4-bipyridyl)ethylene (BPE) probe molecule as well. Figure 5a and b show the Raman spectra measured in air and in water, respectively. Similar to the R6G results, when compared to the dry state measurement, the SERS effect in water was larger by 2.24-fold and 2.43-fold for nanoLCA and NP-nanoLCA, respectively. In addition, the NP-nanoLCA consistently resulted in larger Raman scattering intensity than the nanoLCA did; an average of 18.05-fold larger BPE Raman peak intensity was observed on the NP-nanoLCA when compared to the results from the nanoLCA. Although the EF of the NP-nanoLCA with respect to the nanoLCA for detecting BPE was smaller than R6G detection results, the NP assembly on the nanoLCA surface certainly improved the SERS performance.

We speculate that the pre-coated cysteamine SAM on the NP-nanoLCA surface is the main reason why the enhancement factor was smaller for the BPE than the R6G detection. During the BPE immobilization, the exchange of cysteamine with BPE occurs on the surface of NP-nanoLCA in equilibrium. As the regular nanoLCA does not have cysteamine SAM on its Au surface, dense BPE molecules are adsorbed on the nanoLCA than on the NP-nanoLCA; thus, a smaller number of BPE was detected on the NP-nanoLCA than on the nanoLCA and the EF became smaller for the BPE measurement compared to the R6G quantification. This implies that the actual SERS enhancement on the NP-nanoLCA will be greater than 18.05-fold for detecting the same number of BPE molecules as it is on the nanoLCA surface.

### Importance of plasmon condition matching with the Raman excitation

**Figure 6:**
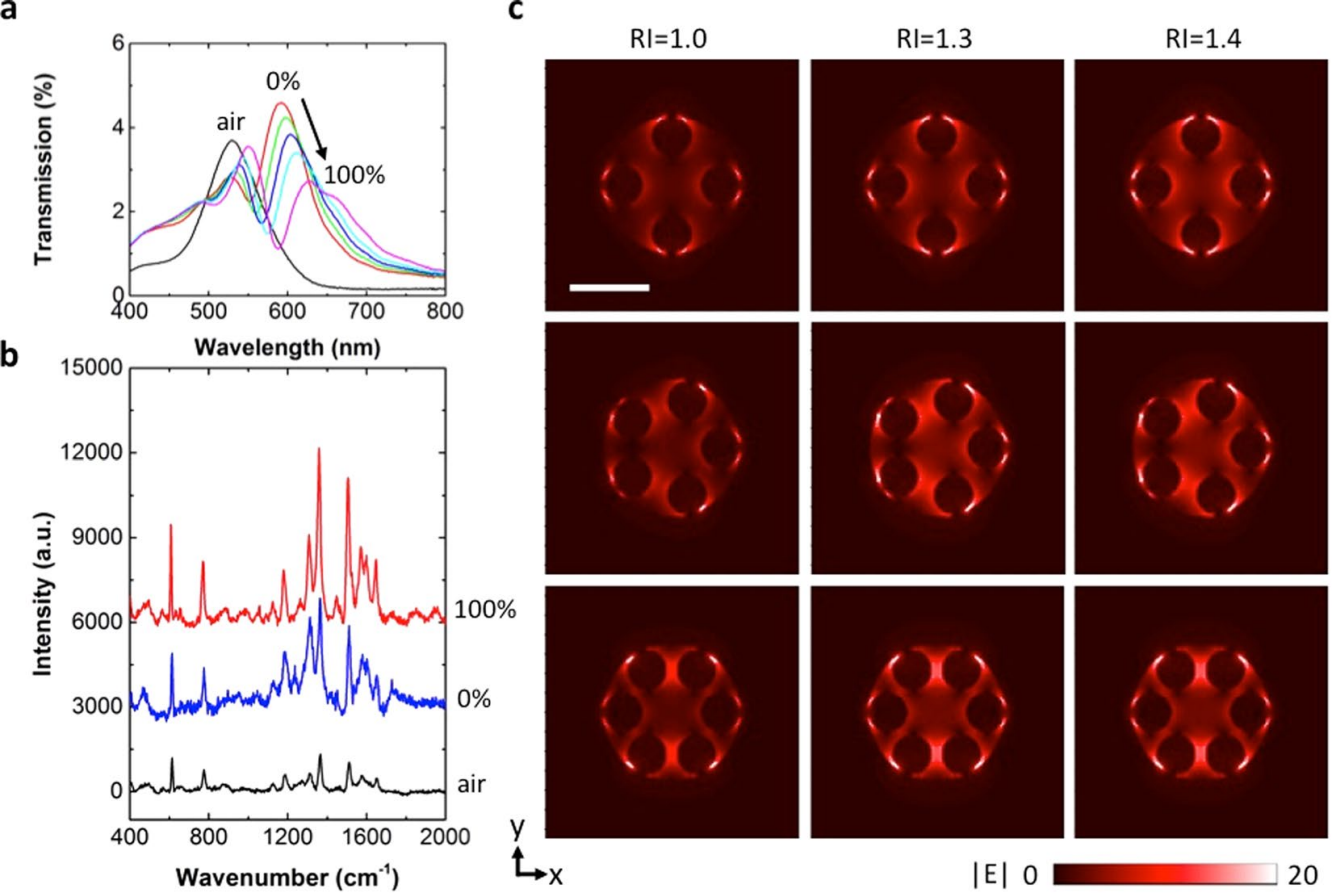
(**a**) The red-shift of the NP-nanoLCA’s surface plasmon resonance wavelength when the surrounding media’s refractive indices increases (i.e., air, 0% glycerol (water), 20% glycerol, 40% glycerol, 60% glycerol, and 100% glycerol). (**b**) Raman spectra of 10 μM R6G on the NP-nanoLCA substrate, measured in air, 0% glycerol, and 100% glycerol. The exposure time was 10 sec. (**c**) Electric field distributions (|E|) of the NP-nanoLCA with varying number of 50 nm NPs on the sidewall. The field distribution shows the stronger electric field confinement with increasing surrounding RIs (from RI = 1.0 to RI = 1.3 and 1.4) as well as denser hot spots generated among the NPs and the nanocup edge. All figures share the same scale bar, which denotes 100 nm.

Both the nanoLCA and the NP-nanoLCA have shown SERS enhancement for the Raman characterization in wet state (i.e., with the sensing medium of water). It was mainly due to the red-shift of the surface plasmon resonance wavelength. The resonance wavelength at ~533 nm shifted to the larger wavelength (i.e., ~590 nm) so that it became closer to the laser excitation wavelength (i.e., 632 nm). The shifted resonance wavelength when measured in water (i.e., 592 nm), on the other hand, was not exactly the same as the excitation wavelength (i.e, 632 nm). Since the resonance wavelength of the nanoLCA or the NP-nanoLCA linearly red-shifts with increasing surrounding RI^20^, the plasmonic response with varying concentration of glycerol solution (0%, 20%, 40%, 60%, and 100%) on the NP-nanoLCA was tracked. As Fig. 6a shows, the resonance wavelength shifted from 592 nm to 628 nm when the surrounding medium on the NP-nanoLCA was changed from 0% glycerol (water) to 100% glycerol. The plasmon resonance wavelength with 100% glycerol was closest to the laser excitation wavelength, which is 632 nm.

To see the effect of the degree of matching between the plasmon resonance and the Raman excitation condition, the Raman spectra of 10 μM R6G were measured on the NP-nanoLCA with the surrounding media of air, 0% glycerol (water), and 100% glycerol. The laser excitation wavelength was consistent to be 632 nm. As the resonance wavelength was located very close to the excitation wavelength, the characteristic Raman peak intensity increased by 2.8-fold and 4.6-fold as the surrounding medium changed from air to 0% glycerol and 100% glycerol, respectively (Fig. 6b). This shows the advantage of using a colorimetric underlying substrate (i.e., nanoLCA) for tuning the SERS EF by a simple replacement of the RI on the sensing surface. Hence, the colorimetric substrate has great potential for maintaining and showing superior SERS performance for detecting a variety of different target molecules, which may have different best Raman measurement conditions.

The numerical FDTD calculation of the electric field distribution (|E|) also verified the importance of matching the plasmon resonance to the laser excitation wavelength (632 nm) in order to achieve effective SERS performance (Fig. 6c). The NP-nanoLCA structures with varying number of NPs (i.e., from 4 to 6 NPs) inside each nanocup were modeled and the electric field distribution (|E|) was calculated. The stronger electric field confinement with increasing surrounding RI (i.e., RI = 1.0, 1.3 and 1.4) on the NP-nanoLCA supports the SERS enhancement by the stronger excitation energy due to increasing localized electromagnetic field. The results also show that the density of hot spots generated at the gap between the NP and the nanocup surface increases with larger number of NPs.

## Discussion

Tunable SERS EF using a colorimetric plasmonic substrate was demonstrated through interfacial dielectric control on the nanoLCA and the NP-nanoLCA substrates. The EF was improved (1) by engineering small gaps among plasmons through a cost-effective self-assembly of the NPs along the 3D surface of nanoLCA and (2) by tuning the surface plasmon resonance condition to provide larger molecular excitation energy near the laser excitation wavelength. Through these two approaches, strong and uniform SERS performance was achieved on a stable Au plasmonic SERS substrate.

A simple and cost-effective method for strong and uniform SERS on a colorimetric plasmonic substrate was accomplished by the self-assembly of NPs along the 3D surface of nanoLCA structure. The sub-1 nm separation through the linker molecule between the NP and the nanoLCA substrate confines strong electromagnetic field; thus, more photons are involved in exciting the molecules and so does the Raman scattering. We demonstrated that the 3D NP arrays amplified Raman signals more than the 2D NP arrays on a thin film. Larger hot-spot density by the plasmon coupling through the assembly of NPs on the 3D nanoLCA substrate improved the SERS performance. An average of 60.94-fold Raman intensity enhancement was observed for R6G detection on the NP-nanoLCA compared to the original nanoLCA substrate. Additional improvement of the SERS performance for the detections of R6G and BPE was achieved by tuning the plasmon resonance wavelength closer to the laser excitation wavelength.

The colorimetric properties of (NP-)nanoLCA substrates facilitated the plasmon resonance tuning through the control over the surrounding media’s RI. Largest SERS EF was obtained when the plasmon resonance wavelength was close to the Raman excitation wavelength. For NP-nanoLCA, larger SERS EF was obtained in the wet state measurement with the plasmon resonance wavelength close to the 632 nm Raman excitation wavelength. Larger SERS EF in wet state measurement, achieved by the colorimetric properties of the NP-nanoLCA, has huge benefit to biomedical applications, as it enables more sensitive detection of target molecules in a complex liquid media, such as cell culture media or solutions, without a need of drying the solvent. Given this tunability of the plasmon resonance wavelength without changing its structure, the NP-nanoLCA substrate can also serve consistently good SERS performance for a variety of chemical detections regardless of the Raman excitation condition that each chemical may require.

## Methods

### Fabrication of NanoLCA and NP-nanoLCA

The nanoLCA and the NP-nanoLCA were prepared to evaluate the SERS performance. The nanoLCA substrate was fabricated by the replica molding technique with a mold, which is composed of nanopillar arrays. The laser interference lithography was implemented to pattern the square array of photoresist islands. The subsequent etching process resolved the nanopillar arrays on quartz mold. Curing a photopolymer (i.e., NOA61) on the mold by exposing it to the ultraviolet light for 1 minute resulted in a polymeric substrate with nanohole array structure. Depositing a thin 90 nm Au layer followed by the 9 nm Ti adhesion layer deposition on the polymer substrate resulted in achieving plasmonic effects on this nanocup array structure^23^.

The NP-nanoLCA was fabricated by self-assembling linker molecules, which have both amine and thiol functional groups at each end. Cysteamine was self-assembled on the Au nanoLCA surface through thiol-Au bonding. The concentration of cysteamine was 10 mM in ethanol. After rinsing the unbound cysteamine molecules, the cysteamine SAM was formed on the Au nanoLCA. Incubating 50 nm Au NP solution (2.0 × 10^11^ particles/mL, purchased from Ted Pella) on this cysteamine-Au nanoLCA substrate for 24 hours resulted in a SAM of Au NPs on the substrate. The NP-nanoLCA was prepared by rinsing the substrate with water and blowing the residual liquid by the nitrogen flow.

### Raman measurements

The probe molecules used in the experiments were R6G and BPE. Each chemical was purchased from Sigma-Aldrich. 2 μL of 10 nM, 100 nM, 1 μM, 10 μM and 100 μM R6G were dropped on the plasmonic substrates. Prior to the Raman measurement, the solvent was evaporated. In order to avoid any coffee-ring effect, the devices were placed on a hot plate with the temperature of 75 °C until the solvent is completely evaporated. On a separate substrate, a monolayer of BPE was assembled by incubating 5 mM BPE solution (in ethanol) for 24 hours. The SAM of BPE on the Au nanoLCA or Au NP-nanoLCA was formed through Au-pyridyle nitrogen bonding between the Au surface and the BPE molecule.

The Raman signals from these probe molecules were detected by Horiba confocal Raman instrument with 632 nm laser source. The laser power was 120 μW and 20 × objective lens (numerical aperture = 0.4) was used. In order to test the influence of the surrounding RI on the SERS substrate, Raman spectra were measured both in air and in water on the nanoLCA. The same measurement condition was used for collecting Raman spectra of the same target molecules on the NP-nanoLCA.

### Transmission measurement

Transmission spectra were collected using BioTek Synergy HTX microplate reader. The spectra were normalized to the intensity of the blank well (empty well); therefore, the spectra were in % transmission. We confirmed that the transmission spectra and the peak position obtained by this microplate reader and the spectra obtained by Cary 5 G spectrometer were identical^24^. For the transmission measurement with liquid interface, 200 μL of each solution was dropped on the (NP-)nanoLCA substrates that were attached on the bottom of the microplate wells.

### FDTD simulations

The 3D nanoLCA and the NP-nanoLCA models were built and simulated using the FDTD simulation software (Lumerical Solutions, Inc. Vancouver, Canada). The nanoLCA model had the same geometry as the actual nanoLCA substrate: the nanocup diameter on top surface was 200 nm, the sidewall angle of the cup was 85 degrees, the depth of the cup was 500 nm, and the periodicity of the cup was 320 nm. The NP-nanoLCA models had the same nanoLCA structures but with additional four, five, six, seven and eight 50 nm-diameter NPs inside of each nanocup. The electric field distributions in RI = 1.0, 1.3, and 1.4 were calculated at each resonance wavelength.

### Data availability

Most datasets generated or analysed during this study are included in this published article and its Supplementary Information files. The datasets generated during and/or analysed during the current study are available from the corresponding author on reasonable request.

## Electronic supplementary material

Supplementary Information
