# Supplementary material for: 3D Plasmon Coupling Assisted Sers on Nanoparticle-Nanocup Array Hybrids
Source: Sci Rep. 2018 Feb 14;8:3002. doi: 10.1038/s41598-018-19256-7 (PMC5813092; doi:10.1038/s41598-018-19256-7)
Supplement: Supplementary file 1 — Supplementary Information [file 41598_2018_19256_MOESM1_ESM.pdf]

Supplementary Information for

3D PLASMON COUPLING ASSISTED SERS ON NANOPARTICLE-NANOCUP ARRAY HYBRIDS

Sujin Seo,[1, 3] Te-Wei Chang,[2, 3] and Gang Logan Liu[2, 3]*

[1]Department of Materials Science and Engineering, University of Illinois at Urbana-Champaign, Urbana, IL 61801, USA
[2]Department of Electrical and Computer Engineering, University of Illinois at Urbana-Champaign, Urbana, IL 61801, USA
[3]Micro and Nano Technology Laboratory, University of Illinois at Urbana-Champaign, IL 61801, USA
*loganliu@illinois.edu

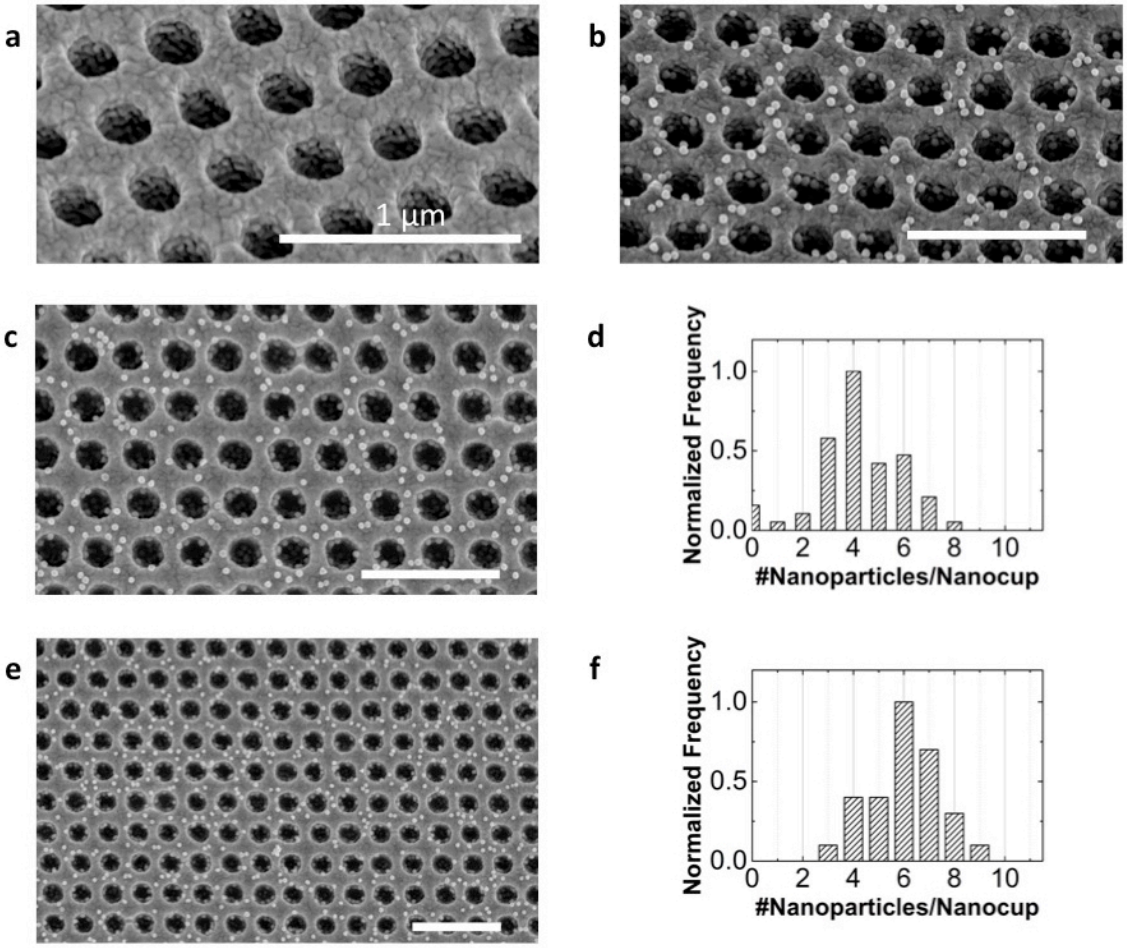

Fig. S1. Scanning electron microscope (SEM) images of (a) nanoLCA and (b, c, e) NP-nanoLCA substrates. Image (a) and (b) are 30° tilted view and image (c) and (e) are top view. (d, f) Histogram of the number of NPs per nanocup for substrate (c) and substrate (e). The average number of NPs is (d) 4.264 NPs/nanocup and (f) 5.8 NPs/nanocup. The scale bar represents 1 μm.

The SEM condition for the images shown in the paper and the supporting information is identical, measured using Hitachi S-4800 with the accelerating voltage of 7kV.

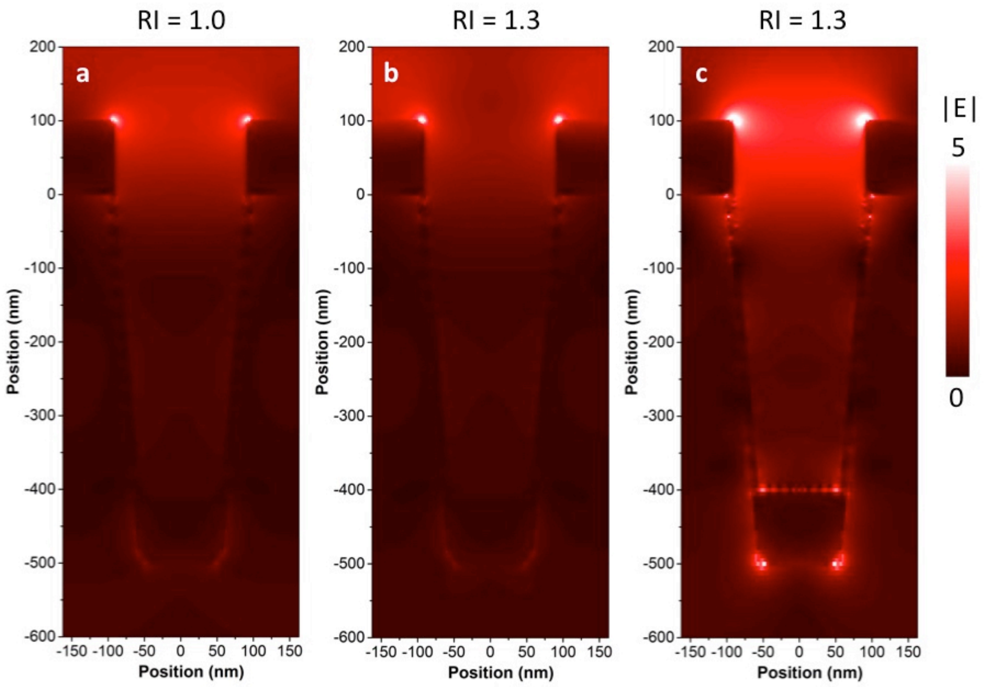

Fig. S2. The plasmon resonance mode of the nanoLCA and the NP-nanoLCA at the transmission peak positions is studied by numerically calculating the electric field distribution (|E|). (a) In RI=1.0, there is a single plasmon resonance peak at 532.5 nm. (b, c) In RI=1.3, on the other hand, there are two plasmon resonance modes (or transmission peaks) at (b) 532.5 nm and (b) 607.5 nm. Based on the field profile, (a) and (c) represent typical localized surface plasmon resonance (LSPR) mode; however, (b) shows the resonance mode of the surface plasmon polariton (SPP). In general, the scattering is mostly governed by the LSPR for the extraordinary transmission phenomena of the plasmonic nanostructure. In addition, the LSPR also plays critical role in enhancing the Raman scattering near this strong electromagnetic field region. This is the main reason for choosing the 586 nm peak (or the 592 nm peak for NP-nanoLCA) as a primary plasmon resonance wavelength when discussing with the experimental results of nanoLCA's transmission spectrum.

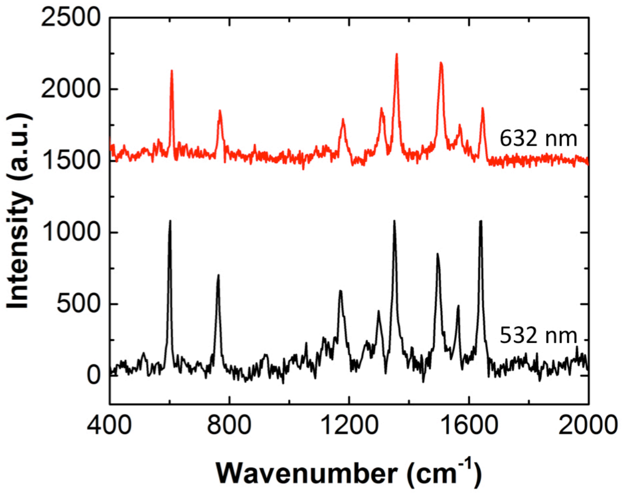

Fig. S3. Raman scattering of R6G measured in air with two excitation wavelengths, 532 nm and 632 nm. As the surface plasmon resonance wavelength of the nanoLCA is 536 nm in air, stronger light scattering from the nanostructured surface increases the chance of light absorption by the nearby molecules. Raman peak intensity at 1358 cm^{-1} was increased by 145.1% with 532 nm laser excitation wavelength.

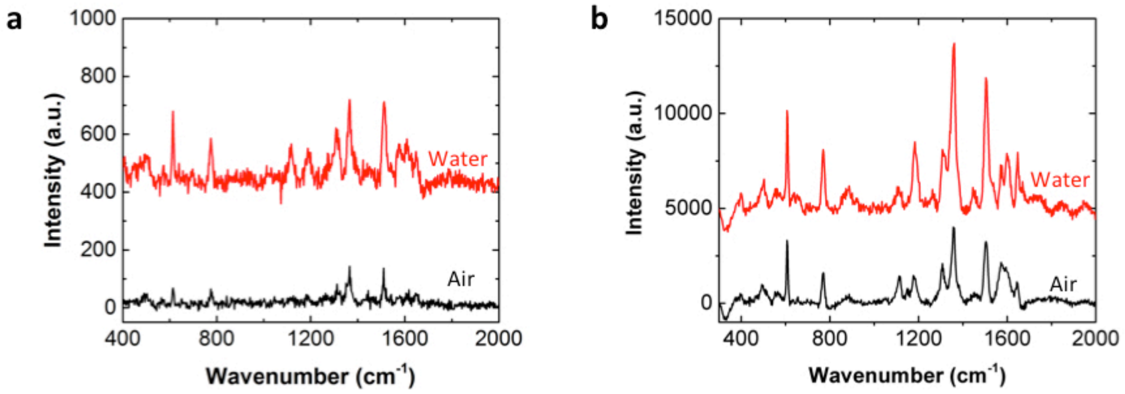

Fig. S4. Raman intensity measured on (a) nanoLCA and (b) NP-nanoLCA. Each measurement was conducted in dry state (air) and in wet state (water). When replacing the sensing media from air to water, 2.16-fold intensity increment was observed for the nanoLCA (a) and 2.17-fold enhancement was observed for the NP-nanoLCA.

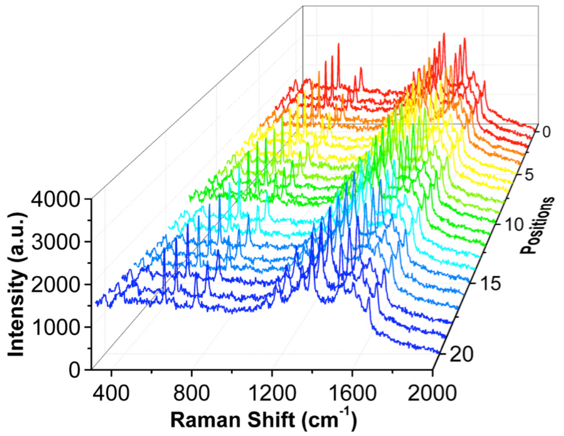

Fig. S5. The Raman scattering spectra from 20 different regions on the 5 mm by 5 mm area on the NP-nanoLCA substrate.

When we consider the laser spot size, the Raman signals were collected from at least 280 nanocups. This averages random SERS signals measured from each individual nanocup with different NP number or different hot spot density.

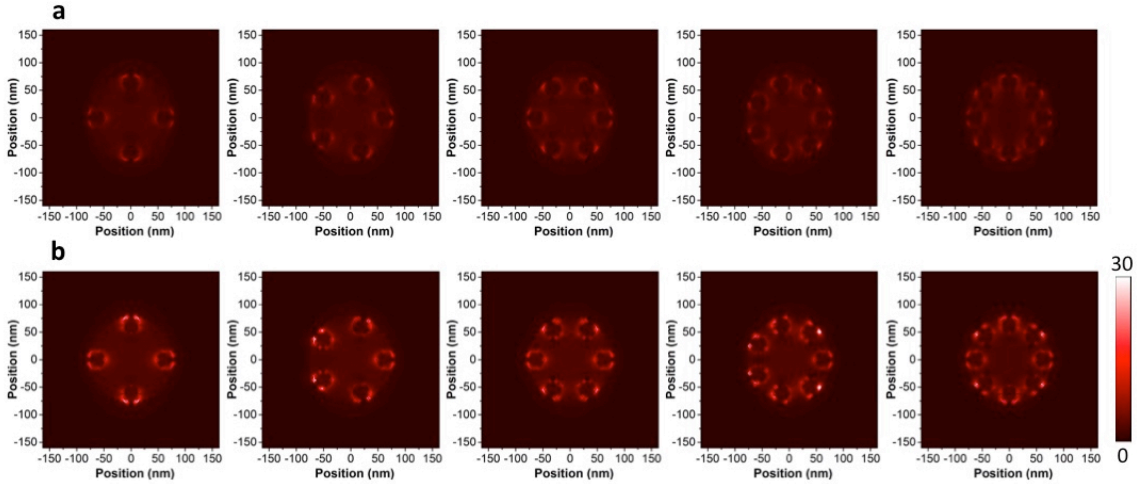

Fig. S6. Electric field distributions (|E|) of the NP-nanoLCAs with different number of NPs collected (a) in 1.0 RIU and (b) in 1.33 RIU. Each field distribution shows field confinement at each surface plasmon resonance wavelength.

The numerical analysis to identify the electric field distributions on the nanoLCA and the NP-nanoLCA was performed using the FDTD simulation. The overall electric field intensity from the results from the surrounding RI of 1.0 RIU (Fig. S5a) was smaller than those results collected in 1.33 RIU (Fig. S5b). For instance, the maximum electric field intensity of the NP-nanoLCA with eight NPs in the 1.33 RIU was 2.87-fold larger than the one in 1.0 RIU. The same comparison for the NP-nanoLCA with four NPs resulted in the 2.34-fold larger electric field intensity in water than in air. The larger electric field confinement or stronger hot-spot intensity in 1.33 RIU than in 1.0 RIU explains why better SERS performance was observed in wet-state than in dry-state for detecting both R6G and BPE. In addition to the electric field distribution, the smaller gap between the laser excitation wavelength (632 nm) and the plasmon resonance wavelength in water (close to 600 nm) supports larger SERS signal in wet-state.

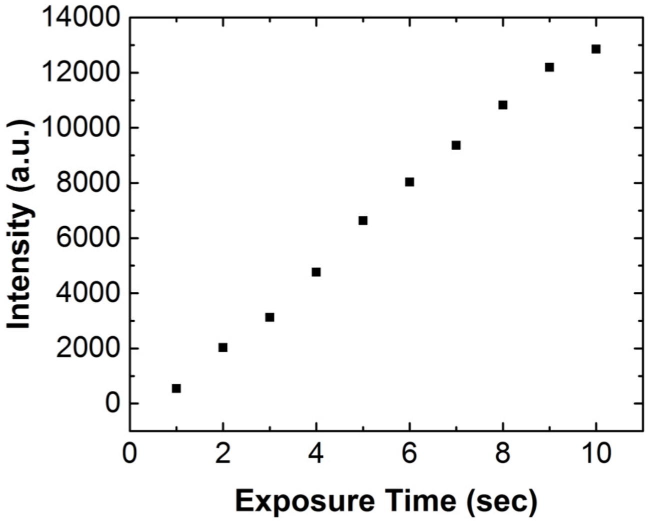

Fig. S7. The R6G Raman intensity at 1357 cm^{-1} linearly increased when the exposure time increased from 1 sec to 10 sec. This linear relationship was implemented when calculating the SERS enhancement factor.

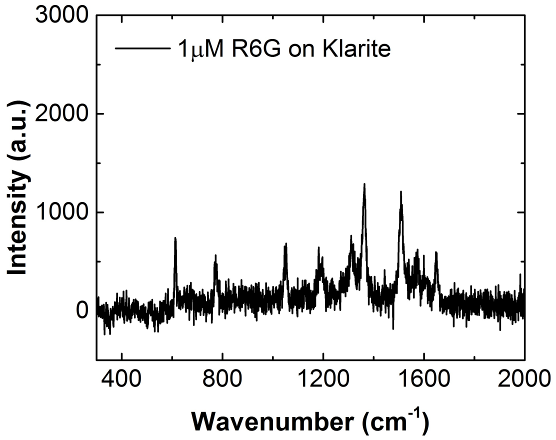

Fig. S8. The Raman spectra of R6G on Klarite commercial SERS substrate.

Enhancement factor calculation

In order to calculate the enhancement factor of the nanoLCA and the NP-nanoLCA substrate, the Raman spectra of 1 μM R6G was measured on a commercial SERS substrate, Klarite, and compared the Raman intensity at the same peak (1357 cm^{-1}). The measurement condition on the Klarite substrate was 211 μW laser power and 10 sec exposure time. The following equation shows how the enhancement factor (EF) was calculated:

$$EF = \frac{I_{SERS}}{I_{Raman}} \times \frac{N_{Raman}}{N_{SERS}} \times \frac{P_{Raman}}{P_{SERS}} \times \frac{T_{Raman}}{T_{SERS}}$$

The Raman intensity on the NP-nanoLCA for measuring 100 nM R6G was 2249.847 with the laser power of 120 μW. In contrast, the intensity on Klarite substrate for measuring 1 μM R6G was 1289 with the laser power of 211 μW. When considering all these parameters, the calculated EF of the NP-nanoLCA was 30.69-fold larger than that of Klarite. The reported EF of Klarite with respect to the bulk R6G probe was approximately 2.8×10^5. The estimated EF of the NP-nanoLCA using this information is 8.42×10^6. This value is not as large as the recent SERS reports, since the underlying substrate was not meant to design to achieve the optimum SERS performance; however, unlike the existing SERS substrates, the nanoLCA has the colorimetric properties, which is induced by the sensitive surface plasmon resonance response to the surrounding refractive index variation. Using this plasmonic substrate with the extraordinary transmission effects that enables colorimetric properties, we verified (1) the positive influence of the additional plasmonic nanoparticle assembly on the SERS improvement and (2) the importance of plasmon resonance condition matching with the Raman excitation condition.
